# Preanalytical classical and alternative complement pathway activity loss

**DOI:** 10.11613/BM.2019.030701

**Published:** 2019-08-05

**Authors:** Koen O.A. Vercauteren, Stijn Lambrecht, Joris Delanghe

**Affiliations:** Department of clinical chemistry, Ghent University Hospital, Belgium

**Keywords:** preanalytical phase, alternative and classical complement pathway, complement haemolytic activity assay, complement C3, complement C3d

## Abstract

**Introduction:**

Complement functional analyses provide insight into the integrity of the entire complement reaction cascade. These tests are suitable for investigating suspected complement deficiencies. Falsely reduced test outcomes may result from preanalytical instabilities of individual complement components. To generate rationale for this or potential alternative practices, this study aimed to extend the knowledge on the preanalytical stability of widely used tests to screen the complement system. We assessed the influence of time, temperature and EDTA on classical (CH50) and alternative pathway (AP50) functional assay test results.

**Materials and methods:**

We used nephelometric (C3d) and immunofixation (C3c) techniques to support the investigation of the preanalytical phase of basic complement system activity tests. Quantitative determination of classical and alternative pathway function was performed with a haemolytic activity assay and a C5b-9 neo-epitope ELISA-based assay respectively. Blood of five healthy volunteers was sampled and complement components allowed to degrade under different conditions.

**Results:**

CH50 and AP50 remain stable for approximately one week in serum samples incubated on ice. CH50 activity decreased almost twice as fast in EDTA plasma compared to serum at room temperature. AP50 activity contrastingly, decreased twice as slow in EDTA plasma compared to serum at room temperature.

**Conclusion:**

Serum on ice remains the preferred specimen for functional complement analyses. In the absence of serum transported on ice, serum kept at room temperature (not exceeding 24h) is suitable for classical and alternative pathway analyses. For alternative pathway analyses specifically, the C3-stabilising effect of EDTA allows for the extended use of EDTA plasma (not over 4 days). In these conditions, at least 85% of baseline complement activity remains.

## Introduction

Primary immunodeficiencies (PID) are a group of relatively rare conditions that are of importance in clinical medicine. Global prevalence of PID is estimated to be a little less than 0.1% (*1*). In PID assessment, exploration of the complement system is of major importance (*2*-*4*). Laboratory complement analysis allows for a more precise differential diagnosis (and critical monitoring of complement-targeted therapy) (*5*). Besides playing vital roles in host defence, inappropriate complement activation causes inflammation (*6*).

The complement system can be activated by three different mechanisms. Besides the classical pathway, also the alternative pathway and the mannose-binding lectin pathway are able to activate the complement system (*3*, *7*). In the clinical laboratory, these three pathways can be explored by assessing total haemolytic (or classical) complement activity (CH50), the alternative pathway activity (AP50) and mannose-binding lectin activity (MBL) (*8*-*10*).

Most complement proteins are known to be brittle structures, vulnerable to degradation (by *in vitro* activation). Therefore, it is generally recommended to cool blood samples for complement analysis as quick as possible to prevent complement factor degradation (*11*, *12*). As analysis of the complement system is mainly carried out in specialised clinical reference laboratories, a well-controlled preanalytical phase is a prerequisite for obtaining reliable results. Whereas it is generally accepted that individual complement factors may degrade rapidly at room temperature, to our knowledge little is known on the preanalytical stability of current functional screening tests for the complement cascade (*11*-*13*).

In the present study we investigated the preanalytical phase of two basic complement system activity tests (CH50 and AP50) in order to generate insights in *ex vivo* complement degradation mechanisms that critically influence complement pathway activities. Next, we aimed to evaluate potential alternative sample types for complement activity testing in case ice-cooled serum is not available or not optimal (*e.g.* suspected cold activation of the complement pathway). Therefore, we assessed the influence of time, temperature and ethylenediaminetetraacetic acid (EDTA) on CH50 and AP50 test results.

## Materials and methods

### Subjects, materials and study design

In order to determine CH50 and AP50 degradation kinetics during preanalytical sample processing (such as sample transportation from external sites), serum and dipotassium EDTA plasma from a single blood draw (BD Vacutainer, Erembodegem, Belgium) was allowed to stand for increasing periods of time on and off ice before freezing. Blood was drawn from five healthy volunteers (inclusion criteria: age 20-30 years, not pregnant, no clinical signs of infections during the last 2 weeks, no use of medication during the last 2 weeks, no history of auto-immune disorders nor immune deficiencies), and kept at room temperature (RT) for 30 minutes. Next, serum and EDTA plasma were separated by centrifugation (2000xg, 8 minutes, 22°C). One fraction of the serum was incubated on ice and another at room temperature, whereas the complete EDTA plasma sample was incubated at room temperature. Before complement analyses, aliquots were frozen at - 20°C on indicated time points relative to serum and plasma separation (0h, 6h, 1d, 2d, 4d and 7d). In an alternative approach, blood samples from two healthy volunteers were allowed to stand at RT and on ice for increasing periods of time (0h, 6h, 1d, 2d, 4d and 7d) before being centrifuged for serum and plasma separation and stored at - 20°C for later complement analyses. The study was approved by the Ghent University hospital Ethics Committee (registration number: EC2015/004) and informed consents signed by all participants.

### Methods

#### CH50 analysis

Quantitative determination of total/classical complement activity (CH50) was performed using the Autokit CH50 (Wako Chemicals, Neuss, Germany) according to the manufacturer’s instructions. The Autokit CH50 is an automated *in vitro* liposome immunoassay (LIA), which was run on a modular-P analyser (Roche Diagnostics, Basel, Switzerland). In short, antibodies in the substrate combine with dinitrophenyl antigens on the liposomes. This antigen-antibody complex activates complements in the patient sample resulting in liposome membrane destruction. This enables the enzyme, glucose-6-phosphate dehydrogenase, contained in the liposome, to react with nicotinamide adenine dinucleotide (NAD) and D-glucose-6-phosphate in the substrate; hence, reducing NAD to NADH. The enzymatic reaction is proportional to the complement activity in the sample that can be monitored by increased absorbance at 340 nm. In house reference values are set between 23 and 63 U/mL. Package insert total precision is 5.7 and 3.2% coefficient of variation (CV) for mean analytical concentrations of 26.9 and 48.3 U/mL respectively (these performance claims were met by in house verification). Measurements were run in singlets.

#### AP50 analysis

Alternative pathway activity (AP50) was determined using the Wieslab (Euro Diagnostica, Malmö, Sweden) Complement system Alternative pathway enzyme immunoassay kit (COMPL AP330). Specific activation of the alternative pathway in the patient sample is driven by LPS (lipopolysaccharide) coated in a microtiter well. This results in the formation of the C5b-9 neoepitope, which is detected by alkaline phosphatase conjugated antibodies. Colorimetric hydrolysis of para nitrophenyl phosphate is proportional to the functional activity of the alternative complement pathway. Package insert reference values are set between 30 and 113%, total precision is 20, 11 and 9% CV for mean analytical values of 16, 48 and 89% respectively (these performance claims were met by in house verification). Measurements were run in singlets.

#### C3d analysis

C3d concentrations were assessed, as a measure of C3 degradation, using an in-house assay, according to Bossuyt and colleagues (*14*). In short, intact C3 was precipitated with polyethylene glycol (PEG) for 2 hours on ice before nephelometric assessment of C3d using polyclonal anti-C3d antibodies (Dako, Glostrup, Denmark). Measurements were run in singlets. In house performance testing showed linearity between 0.01 and 0.2 g/L; clinical cut-off was set at 0.008 g/L (upper level of 95% CI of single measurements on 25 healthy volunteers’ EDTA plasma samples); total precision is 29.8% and 14.9% CV for mean analytical concentrations of 0.007 g/L and 0.056 g/L respectively.

#### C3c immunofixation

Immunofixations were run on a Hydrasys focusing apparatus (Sebia, Ivry, France) using polyclonal anti-C3c antibodies (Siemens, Marburg, Germany). C3c immunofixation was performed to track C3 breakdown/*in vitro* activation. Upon activation, C3 splits into C3c and C3d. Since C3c is part of the native C3 protein, polyclonal C3c antibodies can visualise both components. C3c and C3 can then be distinguished by their differing gel-electrophoretic motilities.

### Statistical analysis

Prism 5.0 was used for graphical presentation and statistical analysis. Non-linear fitting (sigmoidal dose-response function) of normalized-to-maximum C3d and AP50 values to logarithmically transformed time-values was used to visualise the time-dependent inverse relationship between C3d and AP50. Standard linear regression analysis was performed to visualise the inverse correlation between C3d and AP50. The Spearman nonparametric test was used to test correlation significance between C3d accumulation and AP50 degradation.

## Results

**Figure 1:**
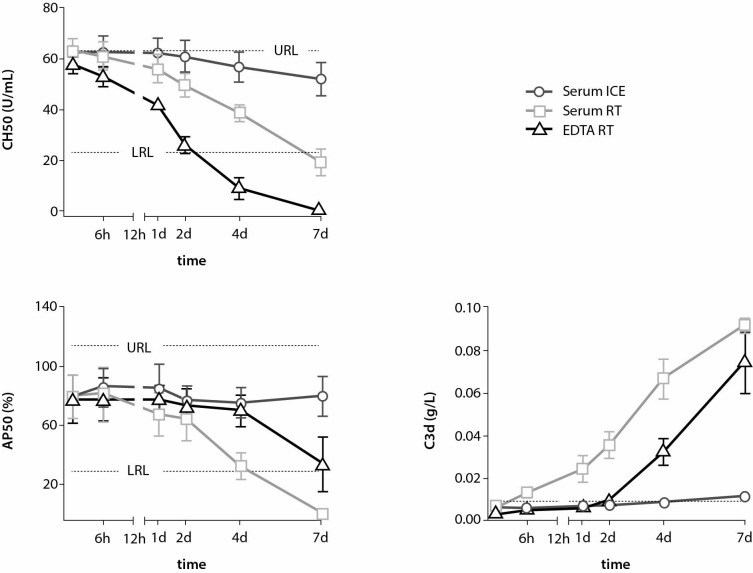
CH50 (top left panel), AP50 (bottom left panel) and C3d (bottom right panel) kinetics in Serum on ice (dark grey) vs. RT (light grey) and EDTA plasma at RT (black). Symbols and error bars represent the mean and standard error of mean of five healthy volunteers. Reference intervals and cut-off are indicated as dotted lines. RT - room temperature. URL - upper reference limit. LRL - lower reference limit. CH50 - total haemolytic (or classical) complement activity. AP50 - alternative pathway com-plement activity. C3d - nephelometric quantitation of C3 split product, C3d.

Figure 1 shows the mean of CH50 (top panel) and AP50 (middle panel) measurements of five healthy volunteers in serum (on ice and at RT) and in EDTA plasma (at RT) in function of time. Neither CH50 nor AP50 activity seem dramatically influenced by standing on ice for multiple days (recovery of 83% and 100% of baseline activities respectively). In contrast, when serum is kept at room temperature similar activity recoveries are observed after a single incubation day (88% and 85% for CH50 and AP50 respectively) before dropping below the lower reference limit after seven days on average. Furthermore, CH50 activity seems considerably less stable in EDTA plasma compared to serum at room temperature. In EDTA plasma, only 66% of baseline serum CH50 activity remains after one day. Interestingly, AP50 activity seems markedly stabilised in EDTA plasma compared to serum at room temperature. In EDTA plasma, still 88% of baseline serum CH50 activity remains after as long as four days. Accordingly, linear regression analysis of CH50 and AP50 kinetics reveal that CH50 decreased almost twice as fast in EDTA plasma compared to serum at room temperature (T_1/2_ = 2.7 and 5 days respectively), while AP50 decreased twice as slow in EDTA plasma compared to serum at room temperature (T_1/2_ = 7.4 and 3.6 days respectively) (data not shown).

**Figure 2:**
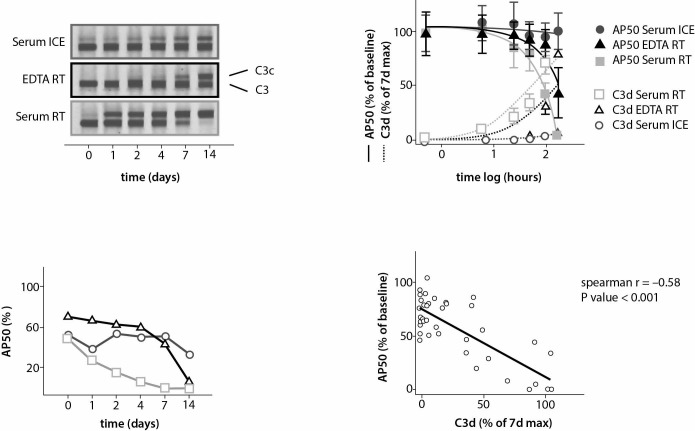
Comparison of AP50 activity (bottom left panel) with C3c formation (upper left panel) by immunofixation using poly-clonal antibodies against C3c in serum (light grey) and EDTA plasma (black) at RT versus in serum on ice (dark grey) from a single healthy volunteer over time. Non-linear fitting of logarithmically transformed time and normalized-to-maximum AP50 and C3d values (upper right panel) in serum on ice (dark grey closed and open circles) vs. RT (light grey closed and open squares) and EDTA plasma at room temperature (black closed and open triangles). Symbols and error bars represent the mean and standard error of mean of three healthy volunteers. Spearman correlation and linear regression analyses (lower right panel): each data point compares individual normalized-to maximum AP50 (Y-axis) and C3d (X-axis) values of all available time points of serum at RT and EDTA plasma from three healthy volunteers. RT - room temperature.

The fastest degradation in serum at RT was observed for AP50 activity. Since complement factor 3 (C3) is a central component of the alternative pathway, we hypothesised that breakdown of C3 could be the root cause of this striking instability. To this end, we performed C3c immunofixation (Figure 2) on blood samples of one healthy volunteer. At baseline, C3 is mostly in its native intact state. Over time, native C3 gets interchanged with its degradation split product C3c. Similar to the observed loss of AP50 activity loss, this C3 degradation process happens fast in serum and is drastically delayed both on ice and in the presence of EDTA.

To gain further insights in the apparent correlation between AP50 activity loss and C3 degradation, we quantitated C3d formation as a measure of C3 degradation in samples from three healthy volunteers (Figure 1, bottom panel). In accordance with AP50 activity, C3d does not form in serum for days when kept on ice, whereas at RT C3d starts to form rather rapidly (Figure 1). Similar to the prolonging effect on AP50 activity, EDTA is able to vastly delay the formation of C3d at RT. Overlaying the AP50 and C3d sigmoidal kinetic curves indeed reveals an inverse relationship (Figure 2). Nonparametric correlation analysis confirms that AP50 activity and C3d formation relate inversely in a statistically significant manner (P < 0.001 and Spearman r = - 0.58).

**Figure 3:**
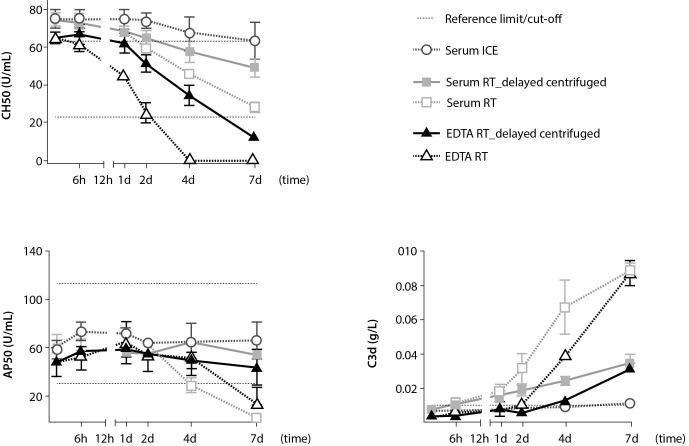
Influence of centrifugation timing on CH50, AP50 and C3d kinetics in serum on ice (dark grey) vs. RT (light grey) and EDTA plasma at RT (black). Symbols and error bars represent the mean and standard error of mean of two healthy volunteers. Kinetics in serum (squares) and EDTA plasma (triangles) when centrifugation is performed before incubation (open symbols connected by dotted lines) compared to centrifugation after indicated incubation times (filled symbols connected by solid lines). Reference intervals and cut-off are indicated as dotted lines. RT - room temperature. AP50 - alternative pathway complement activity. CH50 - total haemolytic (or classical) complement activity.

Finally, to determine the effect of delayed centrifugation on pre-analytical stability of complement pathways, we assessed the stability of CH50 and AP50 activity in serum and plasma samples of two volunteers that were only centrifuged after controlled incubations. In this limited sample size, delay of centrifugation further increased CH50 and AP50 activity stabilities (Figure 3).

## Discussion

Previous research has focused on kinetic follow up of complement degradation by analysing selected individual complement factors, their split products and activation biomarkers (*11*-*13*). In here, we translate a similar idea to the activity of the entire pathway by using functional assays. Individual complement factor follow up of C3(d) was performed essentially to describe the potential mechanism of AP50 instabilities.

The present study demonstrates that CH50 results remain clinically acceptable for 24 hours in serum even at RT, while its stability worsens in the presence of EDTA. EDTA is a chelator of magnesium and calcium that both play key roles in the classical complement pathway, and conformational stability of the C1 (subcomponent) complex (C1q, C1r and C1s) in particular (*15*). The essential role of calcium in this context has more recently been detailed by structural investigation of C1 subcomponent interactions (*16*). EDTA could indeed induce conformational changes of C1 subcomponents; hence, negatively affecting CH50 stability. In contrast, AP50 (in which C1 subcomponents do not play a role) appeared to be considerably stabilised by the presence of EDTA. It has to be noted that MBL functional analyses were not included in this study. However, since the calcium-dependent interaction mechanism of the C1 subcomponents is conserved in the interaction between MBL and MBL-associated serine proteases (MASP, the serine proteases of the lectin pathway activation complexes), a destabilising effect of EDTA is to be expected in functional MBL assays as well (*16*).

Detailed analysis of the breakdown kinetics of AP50, the most temperature-sensitive pathway in serum examined, revealed a strong parallelism between the loss of AP50 activity and the generation of the C3 breakdown products C3c and C3d. In agreement with earlier observations, the rate of C3c and C3d formation (as measures of C3 degradation by *in vitro* complement activation) is strongly temperature dependent in serum and dramatically decreases in the presence of EDTA (*11*, *12*). We observe that C3 degradation significantly correlates with AP50 activity decrease. Therefore, the preanalytical stability of AP50 activity seems predominantly determined by the ability to stabilise (or prevent the *in vitro* activation of) complement factor 3, the central component of this pathway.

Optimal preanalytical conditions differ between the studied assays. These insights may facilitate the collection and transportation of samples used for complement pathway screening. In daily routine, centrifugation of blood samples can be delayed. Although assessed in only 2 out of 5 included volunteers, we observe that delay of centrifugation rather added to CH50 and AP50 activity stabilities. This suggests that the centrifugation process as such can stimulate *in vitro* complement activation.

**Figure 4:**
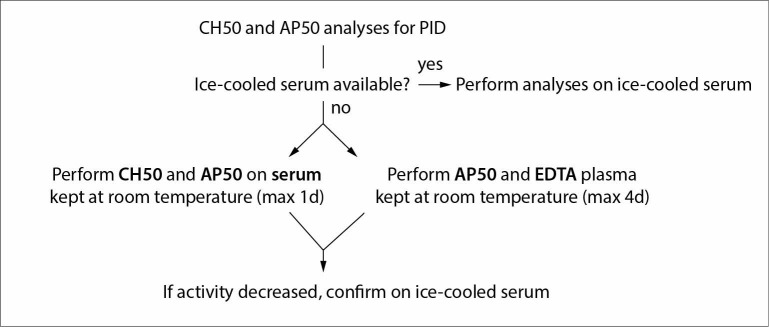
Proposed testing algorithm for CH50 and AP50 analyses.

Altogether, the data generated in here support the testing algorithm proposed in Figure 4. Serum on ice remains the preferred specimen for functional complement analyses. Serum kept at RT (not exceeding 24h) is a suitable alternative for AP50 and CH50 analyses. Moreover, shipment at RT may be a valuable alternative in cases of suspected cold activation of the complement cascade (*17*). EDTA plasma can still be used for AP50 analyses after prolonged standing at RT (not over 4 days old).

Finally, it should be mentioned that we did not validate our findings using functional assays other than those mentioned in here. In addition, the package insert of the used AP50 (and CH50) kit does not declare that EDTA plasma is supported as a specimen for this assay (*18*, *19*). Therefore thorough in-house validation is a prerequisite before using EDTA plasma as a specimen for the latter assay. Another study limitation is the limited number (N=5) of exclusively healthy volunteers. Indeed, strong baseline *in vivo* activation (*e.g.* pathologies involving complement consumption) likely increases *ex vivo* activation and complement degradation. Therefore, we propose to always confirm decreased complement activities using serum samples on ice.
